# Retrieval of Semantic-Based Inspirational Sources for Emotional Design

**DOI:** 10.1155/2018/4685187

**Published:** 2018-11-08

**Authors:** Jian Du, Yan Li, Jinlong Ma, Yan Xiong, Wenqiang Li

**Affiliations:** School of Manufacturing Science & Engineering, Sichuan University, Chengdu, China

## Abstract

In the conceptual design stage, inspirational sources play an important role in designers' creative thinking. This paper proposes a retrieval method for semantic-based inspirational sources, which helps designers obtain inspirational images in the conceptual design stage of emotional design. The core principle involves solving the designer's own deficiencies in associations and limited knowledge, by bridging the “semantic gap” faced by designers when they use Kansei words for inspirational sources. This method can be divided into two aspects: (1) based on the semantic richness of Kansei words, the first part describes how a lexical ontology for Kansei words called KanseiNet is constructed and proposes a spreading activation mechanism based on KanseiNet to complete the semantic expansion of Kansei words; (2) the second part describes how, using existing semantic techniques, relevant design website resources are crawled and analyzed, images' context descriptions and Kansei evaluations are extracted, and Kansei evaluation index of inspirational images is established. The KanseiNet for Chinese is first constructed, and the Sources of Inspiration Retrieval System for Emotional Design (SIRSED) is developed. An experiment comparing the existing image retrieval systems with SIRSED proved the latter to be a more comprehensive and accurate way for designers to access inspirational sources.

## 1. Introduction

With the development of the world economy and the improvement of overall living standards, consumption patterns have changed from product-oriented to consumer-oriented [1]. Some studies suggest that products with good emotional designs can attract customers and affect their choices and preferences [2]. Given this trend, research on consumers' emotions about products has been growing rapidly in the past decade [3]. Emotional design has become an important design area [4].

In the conceptual design stage, designers use inspirational sources to stimulate creative ideas, meaning that they consciously use existing designs and other resources as a reference for solving new problems [5, 6]. The inspirational sources provide the designer with cognitive motivation and support divergent thinking in concept generation [7]. Designers need to constantly be inspired by the inspirational sources, which when combined with their own long-term memory and cognitive processing generate creative ideas [8, 9]. Studies have further revealed that image stimuli can promote more creative ideas than text and designers tend to use images as inspirational sources in the design process [10, 11]. However, designers face difficulties in obtaining suitable inspirational sources, and even expert designers have limited knowledge of themselves [12]. During the design process, designers spend 60% of their time on activities related to information gathering [13]. Therefore, recommending appropriate inspirational sources to designers from a larger and complex set of information is essential.

To the best of our knowledge, in the field of emotional design, the information retrieval needs of emotional designers and providing designers with comprehensive and accurate inspirational sources have been seldom focused upon so far. Kansei engineering proposed by [14] is a product development methodology of acquiring and transforming customer affection into design attribute settings with the use of quantitative methods, which is currently the most widely used method in emotional design and has been used in many successful product designs [15, 16]. “Kansei” is a Japanese word that means psychological feelings, sensations, and emotions. The framework of KE encompasses four tasks: definition of the product domain, determination of the dimensions of customer affection, determination of design attributes and attribute options, and evaluation of relations between customer affection and design attributes. Based on Kansei engineering, scholars have used different techniques to develop corresponding emotional design methods, such as genetic algorithms [1], association rules [17], and neural networks [18]. However, the Kansei Engineering studies use images of similar products as inspirational sources, which essentially reflect thinking convergence. Therefore, they are more suitable for improving the design of existing products in terms of emotional experience; when applied to the design of a new product, they limit the designer's exploration of solution space and mostly do not help designers to generate creative ideas in the conceptual design stage.

In this paper, we propose a retrieval method for semantic-based inspirational sources, and based on the proposed method, we construct the lexical ontology of Chinese Kansei words: KanseiNet for Chinese and develop a corresponding image retrieval tool SIRSED to aid designers to obtain inspirational images in the conceptual design phase of emotional design. The core of the tool is a more complete and accurate way for designers to access inspirational sources compared to existing image retrieval methods, which helps increase their efficiency in the information gathering process and supports divergence thinking and is therefore more likely to produce creative ideas.

## 2. Problem Description and Research Method

Emotional design involves a process of mapping from the emotional needs in the customer domain to Kansei design elements in the design domain [19]. In the customer domain, emotional customer needs are described using Kansei words. In the design domain, designers transform customer emotional needs into Kansei design elements. Therefore, emotional design problems are usually expressed using a set of Kansei words. Designers use these Kansei words to search and gather relevant inspirational images. For example, the design of Italy telecom broadband service terminal Alice was based on Kansei words that can express the brand's unique value and search relevant design sketches, extract design rules, and then generate the final design [20]. Westerman [21] found in their study that by searching with Kansei words, designers could find inspirational images and accomplish the design of concept cars.

In the process of using Kansei words to gather inspirational sources from different databases, the problem that designers face is the semantic gap between low-level features used in content-based image searching and high-level semantic features used in querying. As defined by Smeulders et al. [22], the semantic gap is “the lack of coincidence between the information that one can extract from the visual data and the interpretation that the same data have for the user in a given situation.” The semantic gap thus is the discrepancy between the limited descriptive power of the low-level image features and the richness of user semantics.

Kansei words are rich in semantics. The meanings of the Kansei words are complex, multiple, and may differ across contexts. For example, if a design problem is expressed “gentle,” images that have the characteristics of “gentle” may be described using similar Kansei words such as “elegant,” “cultured,” “soft,” and “tender.” Experienced designers can think of other Kansei words with their mastery of semantics and associations. However, even they may encounter situations where their associations are insufficient. Cognitive psychology reveals that the degree of convenience in gathering and searching knowledge from long-term memory is largely dependent on usage frequency. This means that the “frequent use of particular cognitive structures enhances the chronic accessibility of these structures” [23]. An individual tends to adopt “the path of least resistance,” [24] or may be influenced by “cognitive inertia,” [25], which means that designers primarily associate Kansei words only from accessible areas of their memory, since it requires less effort. Moreover, the richness of semantics in Kansei words also causes difficulties for computers in semantic analysis and comprehension. Ward et al. [26] have studied image browsing in the fashion industry, which indicated that in the pattern of example searching, fashion designers want the current image searching system to not only search the images that match keywords, but also to search those that match high semantic features while not matching low-level visual similarities. In general, most current navigation models are more suitable for the searching task when designers have clarity about the images they wish to acquire, but are not suitable to inspire them.

Currently, most image searching methods are based on keyword searching. Keywords in image searching can generally be divided into two types [27]. One corresponds to the identifiable items in the visual content of an image, such as its colour, texture, and certain entity contained in images (a car, a flower). The other corresponds to abstract concepts contained or expressed in images such as time, space, event, and emotion. Advances in image analysis, object detection, and classification techniques help with automatically extracting the first type of keywords in images. In emotional design, designers generally use the second type of keywords to perform image searching. For example, they use Kansei words such as “cute” and “modern.” However, from the perspective of character representation, it is difficult to find such keywords with respect to specific corresponding visual appearance characteristics in images and extracting them from images automatically thus becomes problematic [28, 29].

In order to assist emotional designers with obtaining inspirational sources, compensating for the problem of the designer's limited knowledge and the semantic gap when searching for inspirational sources, a new method of retrieval of semantic-based inspirational sources is proposed, primarily divided into the following two aspects:

Focus on the problem of Kansei words' semantic richness and designer's deficiencies in associations, establish a Kansei word lexical ontology KanseiNet, propose a spreading activation mechanism based on KanseiNet, and accomplish the semantic expansion of Kansei words.

Utilize existing semantic techniques to crawl inspirational images on designer-oriented websites, extract the context description and Kansei evaluations of such images, and establish a Kansei evaluation index of these images.

## 3. Theoretical Background and Relevant Works

### 3.1. Semantic Query Expansion

According to the existing literature, there are two types of semantic query expansion methods: those based on semantic relation/structure and those based on large-scale corpus [30, 31]. Methods based on semantic relation/structure are usually based on existing thesauri/ontologies [32], such as WordNet and HowNet, and domain ontologies such as Gene Ontology. Methods based on corpus use co-occurrence analysis to realize semantic expansion [33]. Co-occurrence usually analyzes the whole document or part of document based on the principle that words that have a large degree of co-occurrence in corpus are ordinarily relevant [30]. Owing to the lack of a label corpus that focuses on Kansei words and their semantic richness, methods based on semantic relations are more suitable for semantic query expansion of Kansei words.

Semantic relations require the use of existing thesauri/ontologies for extraction or identification. So far, many types of semantic thesauri/ontologies have been developed. However, existing thesauri/ontologies face the following limitations in establishing Kansei word's semantic relations:

Little research so far has focused on Kansei words. Although Kansei words are usually adjectives, they contain information related to the customer's emotion forming. Current semantic ontologies only consider Kansei words as adjectives. For example, HowNet only indicates whether a Kansei word tends to be negative or positive and does not detail the word adequately to meet the requirements of emotional design.

Kansei word meanings differ in different domains, and existing semantic knowledge bases offer no semantic component analysis of Kansei words in the emotional design domain. For example, the Chinese Kansei word “好看的(good-looking)” means that a product has a favourable appearance when describing the product in emotional design, its corresponding expansion words can be “pretty,” “beautiful,” and so on. However, in other domains such as describing movies or fiction, it can mean that the plot is complex and exciting, and its corresponding expansion words would be “winding,” “interesting,” and so on. Therefore, it is difficult to use existing dictionaries to engage in semantic expansion of Kansei words to meet the requirements of emotional design.

Conducting semantic reasoning and expansion from structural aspects has its limitations. As highlighted by [34]; lexical repositories such as WordNet do not capture the semantic relationships between concepts. Semantic repositories such as OpenCyc that are developed to capture and represent common sense do not contain semantic relationships (e.g., whether two concepts are synonyms), and HowNet defines and analyzes Kansei words, but does not establish the connection between Kansei words, which makes it difficult to engage in further semantic reasoning.

To solve the above problems, the Kansei word lexical ontology KanseiNet is constructed through conceptual deconstruction of Kansei words and the calculation of the similarity of senses.

### 3.2. Concept Deconstruction of Kansei Words

**Figure 1:**
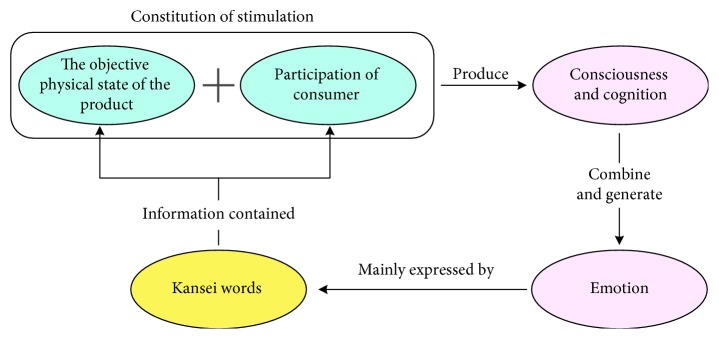
Basic process of generating and expressing customer emotions for products.

Kansei words are verbal elements that can effectively express affective intention when humans express their feelings, such as gentle, terrible, scary, cute, and huge.  Figure 1 presents the basic process of generating and expressing customer emotion in the context of products. In cognitive psychology, external stimulus is considered as the direct cause of human emotion generation [35]. In the product design domain, if a customer is generating emotions over a product, the product should first have a certain objective physical status such as style and colour assortment. Second, customers should engage in certain participative behaviour. For example, if a product can be characterized as “beautiful” but the customer does not see it, then it is impossible to generate corresponding emotions. As a result, the objective physical status of products and participative behaviour of customers combines to form emotion stimulus. Such stimuli are then transferred to sensory receptors and generate feelings, and various types of feelings that are synthetically treated by the brain are processed to generate consciousness and cognition. Next, cognition compares with experience and transforms into emotion, which is finally expressed using Kansei words [36]. This indicates that as Kansei words express human emotions, they also simultaneously convey two types of information, namely, the customer's participating process and the product's physical status. Based on these two types of information, Kansei words can be further analyzed and defined, thus generating semantic concepts of Kansei words.

For expressing the word, structural semantics scientists have proposed a method of semantic componential analysis [37], using sense and sememe to express a word's semantics. Further, HowNet uses sense and sememe to describe Chinese vocabulary in a standardized manner.

- Sense: Sense describes the word's semantics. Each word can be expressed using several senses. Sense is described using a knowledge-expressing language. The words used in this knowledge-expressing language are sememes.
- Sememe: It is the smallest meaning unit used to describe a sense. It is extracted from all words to describe other words, and it is the indivisible basic element.

For a Chinese word ‘男女老少,' the description in HowNet is as follows:

  DEF = {human|人: modifier = {aged|老年}}; {human|人: modifier = {child|少儿}}; {human|人: modifier = {female|女}}; {human|人: modifier = {male|男}}
  “男女老少” has four senses: male, female, aged, and child. “老年” and “少年” are the sememes used to describe the senses.
  Based on an analysis of the two types of information contained in Kansei words and the concept of sense, sememe in structural semantics, a new concept deconstruction method is proposed for Kansei words.

### 3.3. Spreading Activation (SA) Model

The spreading activation (SA) model is a cognitive model used to describe the semantic memory mechanisms of the human brain [38]. The SA technique consists of two components: a semantic network and a SA mechanism [39]. The semantic network includes nodes representing semantic concepts and weighted connections between nodes. After obtaining external stimuli, the corresponding nodes in the semantic network are activated and assigned activation values. According to the SA mechanism, the activation values are passed from the initial activation nodes to the nodes connected with them through the weighted links until the diffusion process is completed. The SA technology simulates the process of human association and has been widely applied in cognitive science, database, AI, biology, information retrieval, etc. [40]. After the rise of knowledge push research, many scholars have applied it to personalized knowledge push in Internet, e-commerce, and social networking, and achieved good results [41, 42].

## 4. Proposed Method

**Figure 2:**
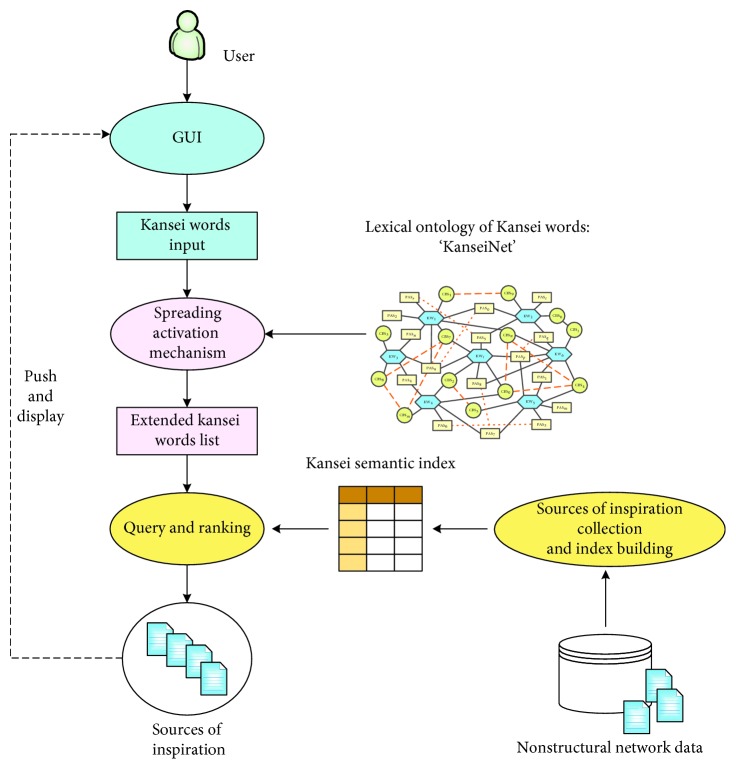
Retrieval method of semantic-based inspirational sources for emotional design.

Based on the discussion so far, in order to assist designers to obtain inspirational sources in emotional design, we need to semantically expand Kansei words and extract abstract emotional concepts expressed in inspirational images. As indicated in Figure 2, the proposed method primarily includes three key parts: 1. KanseiNet, a lexical ontology of Kansei words; 2. KanseiNet-based SA mechanism; 3. Semantic-based Kansei indexing of inspirational images. Each part is described in detail in the following section.

### 4.1. Lexical Ontology of Kansei Words: KanseiNet

As we described in Section 3.2, Kansei words have semantic complexity, and the existing semantic dictionaries are not sufficiently researched on Kansei words, and are not structurally suitable for semantic reasoning and semantic expansion of Kansei words. Therefore, we propose to construct KanseiNet, a lexical ontology of Kansei words. To meet the requirements of semantic expansion in emotional design, KanseiNet's goal is to make up for the shortcomings of the research of existing semantic dictionaries on Kansei words in the following points:

- Deconstructing the meanings contained in Kansei words in the field of emotional design
- Capturing the semantic relationships between Kansei concepts
- Establishing the semantic relationships between Kansei word

HowNet has conducted extensive research on the semantic deconstruction of words and the acquisition of the relationship between concepts. As described in Section 3.3, HowNet deconstructs words into senses and describes them with sememes. The downside is that HowNet failed to interpret the Kansei words more deeply and structurally failed to establish semantic relationships between Kansei words. Therefore, KanseiNet has adopted a similar method of describing words semantically with HowNet. Specifically, the construction steps of KanseiNet are as follows:

- Conceptual deconstruction of Kansei words
- Determination of similar relationships between Kansei senses
- Establishment of the semantic relationships between Kansei words

#### 4.1.1. Conceptual Deconstruction of Kansei Words

Based on the analysis of Kansei words in Section 3.2, Kansei words are deconstructed into two types of senses: product attribute senses and consumers' behaviour senses. Further, Kansei words are described using a two-tuple structure: KW = {PAS, CBS}, where KW represents the Kansei word, PAS represent product attribute senses, and CBS represent consumers' behaviour senses.

*(1) Consumers' Behaviour Senses (CBS)*. CBS describe information about consumer participation behaviour contained in Kansei words. Consumers' participation behaviour forms events, and events are expressed in verbs in language. Therefore, CBS is described using a three-tuple structure: (CBS = (Action, Target, Emotion)). The three sememes in the tuple, Action, Target, and Emotion are defined as follows:

 
*Action*: This sememe represents the consumers' actions related to the event, such as watching, listening, and touching.
 
*Target*: This sememe represents the object of action in the event. In emotional design, the object of action includes the surface of the product, the tone, and so on.
 
*Emotion*: This sememe represents the emotion reflected by the consumers' behaviour sense.

Using the proposed knowledge representation model, the Kansei words “good-looking” and “beautiful” are expressed as follows:

  good-looking = {PAS = {∅}, CBS = {look|看: target = {appearance|外观: host = {product|产品}}, emotion = {joy + surprise}}}
  beautiful = {PAS = {∅}, CBS = {look|看: target = {appearance|外观: host = {product|产品}}, emotion = {joy + surprise}}}

*(2) Product Attribute Senses (PAS)*. PAS describe the information of the objective state of products contained in Kansei words. All things as the host contain attributes, and attributes have a certain value. In the field of emotional design, information about the objective world state of the product is expressed as the product attribute and its attribute value. Therefore, PAS are described using a three-tuple structure: (PAS = (Value of Attribute, Attribute, Emotion)). The three sememes in the tuple, Value of Attribute, Attribute, and Emotion are defined as follows:

 
*Value of Attribute*: this sememe represents the attribute value of the product attribute
 
*Attribute*: this sememe represents the product attribute
 
*Emotion*: this sememe represents the emotion reflected by the product attribute senses

By using the proposed knowledge representation model, the Kansei word “durable” is expressed as follows:

  durable = {PAS = {long|长, Attribute = {usetime|使用时间: host = {product|产品}}, emotion = {trust}},CBS = {use|使用: target = {product|产品}, emotion = {trust}}}}

#### 4.1.2. Determination of Similar Relationships between Kansei Senses

Two senses may have similar relationship though they may be described using different sememes. For example, two user behavioural senses can be depicted as follows:

  CBS_1_ = {use|使用: target = {product|产品}, emotion = {trust + joy}}; CBS_2_ = {carry|携带: target = {product|产品}, emotion = {trust}}.

Carrying a product is also one of the uses of a product. Therefore, CBS_1_ and CBS_2_ can be considered to have a similar relationship.

In order to determine whether a similar relationship exists between senses, we need to calculate the similarity of senses and simultaneously set the similarity threshold *T*_s_. When the similarity between two senses is greater than *T*_s_, the two meanings can be considered to have similar relations.

The calculation of the similarity of senses is based on that of the similarity of sememes. The similarity of sememes is calculated based on the taxonomies of the sememes of KanseiNet.

**Figure 3:**
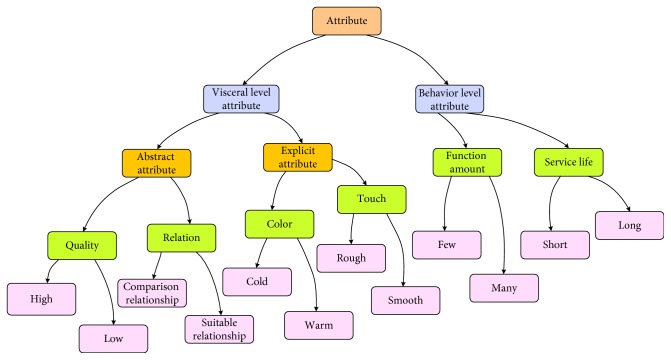
Taxonomies of sememes of KanseiNet (part).

In our research, we deconstructed 215 Kansei words and obtained corresponding sememe sets. These sememes were further classified into taxonomies of sememes of KanseiNet, as shown in Figure 3. Because the attribute value is reflected only by the corresponding attribute, attribute sememes and attribute value sememes were combined in a taxonomy.

Based on existing research, considering the length of the path between nodes and the depth of nodes, the following formula is proposed for calculating the similarity of Kansei sememes:

(1) $$\begin{array}{c} \text{Sim}\left({\text{KS}}_{1},{\text{KS}}_{2}\right)=\frac{\min\left({\text{depth}}_{{\text{KS}}_{1}},{\text{depth}}_{{\text{KS}}_{2}}\right)\times \partial }{\min\left({\text{depth}}_{{\text{KS}}_{1}},{\text{depth}}_{{\text{KS}}_{2}}\right)\times \partial +\text{distance}\left({\text{KS}}_{1},{\text{KS}}_{2}\right)}, \end{array}$$

where min (depth_KS_1__, depth_KS_2__) is the smallest depth of two Kansei sememes nodes in the taxonomies of sememes, and distance (KS_1_, KS_2_) represents the path length between two Kansei sememes. Alpha is an adjustment parameter, which generally takes the path length of a similarity of 0.5.

Further, the similarity of senses is based on the weighted average of the similarity of the Kansei sememes. For PAS, the formula of similarity is calculated as follows:

(2) $$\begin{array}{c} \text{Sim}\left({\text{PAS}}_{1},{\text{PAS}}_{2}\right)=\gamma_{1}{\text{Sim}}_{\text{a}}\left({\text{PAS}}_{1},{\text{PAS}}_{2}\right)+\gamma_{2}{\text{Sim}}_{\text{e}}\left({\text{PAS}}_{1},{\text{PAS}}_{2}\right), \end{array}$$

where Sim_a_(PAS_1_, PAS_2_) represents the similarity of two attribute value sememes, Sim_e_(PAS_1_, PAS_2_) represents the similarity of emotion sememes of two product attribute senses, *γ*_1_, *γ*_2_ are weight parameters, and *γ*_1_+*γ*_2_=1.

For CBS, the following formula of similarity calculation is used:

(3) $$\begin{array}{c} \text{Sim}\left({\text{CBS}}_{1},{\text{CBS}}_{2}\right)=\beta_{1}{\text{Sim}}_{\text{a}}\left({\text{CBS}}_{1},{\text{CBS}}_{2}\right)+\beta_{2}{\text{Sim}}_{\text{ar}}\left({\text{CBS}}_{1},{\text{CBS}}_{2}\right)+\beta_{3}{\text{Sim}}_{\text{e}}\left({\text{CBS}}_{1},{\text{CBS}}_{2}\right). \end{array}$$

Sim_a_(CBS_1_, CBS_2_) represents the similarity of two action sememes, Sim_ar_(CBS_1_, CBS_2_) represents the similarity of two object of action sememes, Sim_e_(CBS_1_, CBS_2_) represents the similarity of emotion sememes of two consumers' behaviour senses, *β*_1_, *β*_2_, *β*_3_ where are weight parameters, and *β*_1_+*β*_2_+*β*_3_.

#### 4.1.3. The Structure of KanseiNet

**Figure 4:**
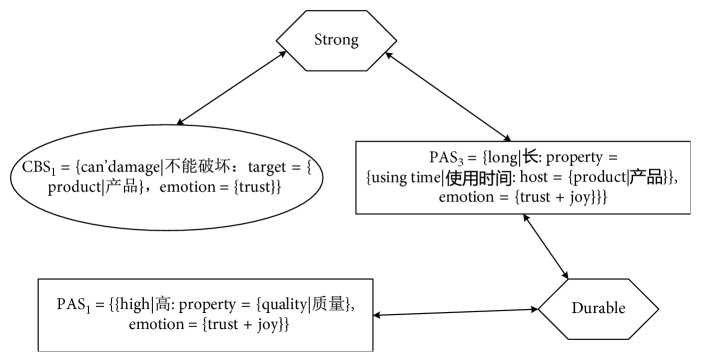
Many-to-many mapping between words and concepts.

KanseiNet is built on the basis of the following features of Kansei words and senses:

1. Classification of senses: In Section 4.1.1, Kansei words were deconstructed into PAS and CBS. These two kinds of senses constitute the knowledge representation of Kansei words.
2. Many-to-many relations: There are many-to-many mapping relations between Kansei words and senses. A Kansei word can be deconstructed into multiple senses, while multiple Kansei words may impart a certain sense. For example, as Figure 4 indicates, the Kansei word *Strong* has 2 senses: CBS_1_ and PAS_2_; meanwhile, the Kansei sense PAS_2_ maps to two Kansei words: *Strong* and *Durable*.
3. Similar relations between senses: In Section 4.1.2, the similarity of Kansei senses is calculated and the similar relationships between Kansei senses are determined.

**Figure 5:**
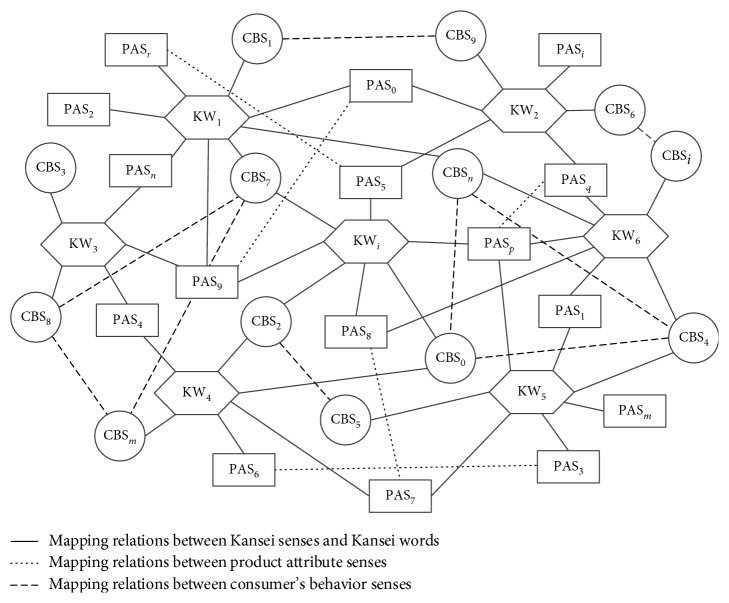
Structure of KanseiNet

By deconstructing the concept of Kansei words, and based on the mapping relationships between Kansei words and Kansei senses and the similar relationships between Kansei senses, the structure of KanseiNet is determined as shown in Figure 5. The purpose of KanseiNet is to establish semantic relationships between Kansei words. As can be seen from Figure 4, there are two types of connections between Kansei words in KanseiNet:

- A Kansei sense establishes the semantic relationship between two Kansei words. That is, two Kansei words have the same Kansei sense. For example, as shown in Figure 4, Kansei word KW_1_ has a product attribute sense PAS_0_; at the same time, PAS_0_ is also a product attribute sense of Kansei word KW_2_. Therefore, in KanseiNet, through PAS_0_, the semantic relationship between Kansei words KW_1_ and KW_2_ is established.
- A pair of similar senses establishes the semantic relationship between two Kansei words. For example, as shown in Figure 4, Kansei word *KW*_7_ has a user behaviour sense CBS_2_, and KW_5_ has another user behaviour sense CBS_5_, and CBS_5_ has a similar relationship with CBS_2_. Therefore, in KanseiNet, through CBS_2_ and CBS_5_, the similarity between the Kansei words KW_7_ and KW_5_ is established.

Generally speaking, KanseiNet puts forward a brand-new concept deconstruction method for Kansei words and establishes semantic relationships between Kansei senses and Kansei words to meet the retrieval needs of designers in emotional design. Compared with existing semantic ontologies or dictionaries, KanseiNet has the following characteristics:

1. In the field of emotional design, a deep conceptual deconstruction of Kansei words is carried out.
2. The semantic relationships between Kansei senses are determined.
3. The connection between Kansei words is established according to the relationships between Kansei words and concepts and between concepts and concepts. This makes it possible to use KanseiNet to carry out semantic reasoning and extension of Kansei words.

Therefore, compared with the existing semantic dictionary, KanseiNet is more suitable for assisting designers to complete the association of Kansei words, to bridge the semantic gap faced by designers in searching the inspirational sources in emotional design.

### 4.2. Semantic Expansion of Kansei Words Based on KanseiNet and SA Model

KanseiNet is further combined with the SA model to assist designers to make associations between Kansei words.

**Figure 6:**
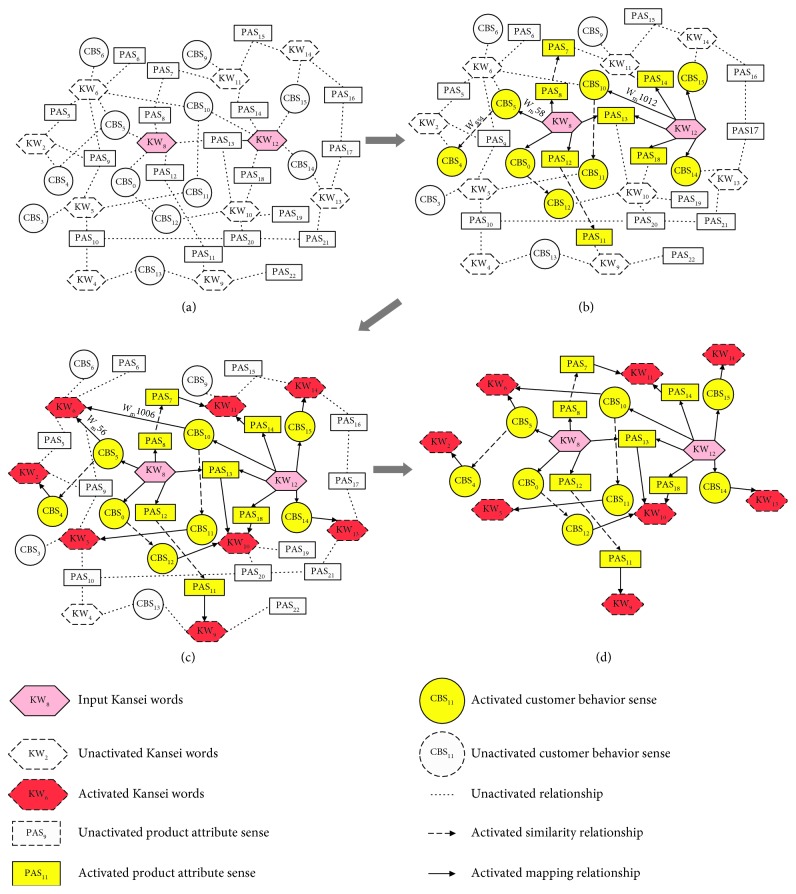
Layered spreading activation process.

Although there is no obvious layer distinction of semantic nodes in KanseiNet, it uses a layer-by-layer process for a SA, as depicted in Figure 6. When a node is activated, its activation value will spread to the neighboring node. The layered spreading activation is unidirectional, including the following four steps:

#### 4.2.1. Activation of Initial Layer

As indicated in Figure 6, designers input Kansei words KW_8_ and KW_12_, following which the corresponding word nodes defined as the initial layer in the KanseiNet are activated. The initial activation value of initial nodes is *p*_*b*_.

#### 4.2.2. Activation of Sense Layer

After the completion of the activation of the initial layer, the next step is to activate the sense layer. There are two types of Kansei sense nodes that are activated in this step:

1. The Kansei sense nodes that form mapping relationships directly with the Kansei word nodes of initial layers, for example, as shown in Figure 6, the Kansei sense CBS_5_ forms a mapping relationship with the Kansei word KW_8_; thus, CBS_5_ is activated in this step.
2. The Kansei sense nodes that form similar relationships with the Kansei sense nodes which form mapping relationships directly with the Kansei word nodes of initial layers, as shown in Figure 6, the Kansei sense CBS_4_ forms a similar relationship with the Kansei sense CBS_5_; meanwhile, the Kansei sense CBS_5_ forms a mapping relationship with the Kansei word KW_8_; thus, CBS_4_ is activated in this step.

Correspondingly, there are two types of relationship weights in this step:

1. *ω*_m_: the weight of mapping relationships between Kansei senses and Kansei words.
2. *ω*_s_: the weight of similar relationships between Kansei senses.

Therefore, for activated Kansei sense node *n*_*s*_ in the sense layer, the calculation of the activation value *p*_*s*_ of node *n*_*s*_ is defined as follows:

(4) $$\begin{array}{c} p_{s}=\overset{n}{\underset{u}{\sum }}p_{u}\omega_{mu}+\overset{m}{\underset{j}{\sum }}p_{j}\omega_{sj}, \end{array}$$

where *p*_*s*_ is the total inputs of the node *n*_*s*_, *p*_*u*_ is the activation value of the node *n*_*u*_ which forms mapping relationship with node *n*_*s*_, *ω*_*mu*_ is the weight of the mapping relation between *n*_*u*_ and *n*_*s*_, *p*_*j*_ is the activation value of the node *n*_*j*_ which forms similar relationship with node *n*_*s*_, and *ω*_*sj*_ is the weight of the similar relation between *n*_*s*_ and *n*_*j*_. For example, as shown in Figure 6, CBS_5_ only forms a mapping relationship with the activated Kansei word KW_8_ in the initial layer. The weight between CBS_5_ and KW_8_ is *w*_m58_, and the activation value of KW_8_ is *p*_*b*_. Therefore, the activation value of CBS_5_ is *P*_CBS5_ = *w*_m58_*p*_*b*_, while CBS_4_ only forms a similar relationship with CBS_5_ and the weight between CBS_5_ and CBS_4_ is *w*_s58_, so the activation value of CBS_4_ is *P*_CBS4_ = *w*_m58_*p*_*b*_*w*_s58_.

#### 4.2.3. Activation of Expansion Layer

The activated nodes of the sense layer in Step 2 further spread to Kansei word nodes in the expansion layer, as shown in Figure 6. For Kansei word node *n*_*c*_ in the expansion layer, the activation value *p*_*c*_ of node *n*_*c*_ is calculated as follows:

(5) $$\begin{array}{c} p_{c}=\overset{h}{\underset{r=1}{\sum }}p_{r}\omega_{mc}, \end{array}$$

*p*_*c*_ is the total inputs of the node *n*_*c*_, *p*_*r*_ is the activation value of the node *n*_*r*_ in the sense layer which forms mapping relationship with node *n*_*c*_, and *ω*_*mc*_ is the weight of the mapping relation between *n*_*c*_ and *n*_*r*_. For example, as shown in Figure 6, the Kansei word KW_6_ has a mapping relationship with the Kansei senses CBS_4_ and CBS_10_, respectively. The activation value of CBS_10_ is *w*_m1012_*p*_*b*_ by formula (4). Therefore, according to formula (5), the activation value of KW_6_ can be obtained as follows: *P*_KW4_=*w*_m56_*w*_m58_*p*_*b*_+*w*_m1006_*w*_m1012_*P*_*b*_.

#### 4.2.4. Determination of Activation Condition and Termination Condition

The activation and termination conditions affect the spreading process, and they can control the spreading process and obtain optimal results. SA applied to KanseiNet aims to complete the semantic expansion of Kansei words entered by designers. The structure of the KanseiNet ensures that all the Kansei words related to Kansei words of the initial layer exist in the expansion layer; therefore, Spreading process is terminated when the activation value of initial layer spreads to the expanded Kansei words, and all the word and sense nodes that are spread are considered to be activated. A node network formed by all activated nodes can be obtained after spreading activation, as indicated in Figure 6.

Further, we calculate the activation values of the expanded layer's Kansei words using formulas (4) and (5), sort the Kansei words based on the calculated activation values, and complete the semantic expansion of the input Kansei words.

### 4.3. Semantic-Based Kansei Indexing of Inspirational Images for Emotional Design

In order to collect images that can stimulate designers' inspiration and extract Kansei evaluations expressed by the description of these images, a semantic-based Kansei indexing of inspirational images for emotional design is proposed in this paper.

**Figure 7:**
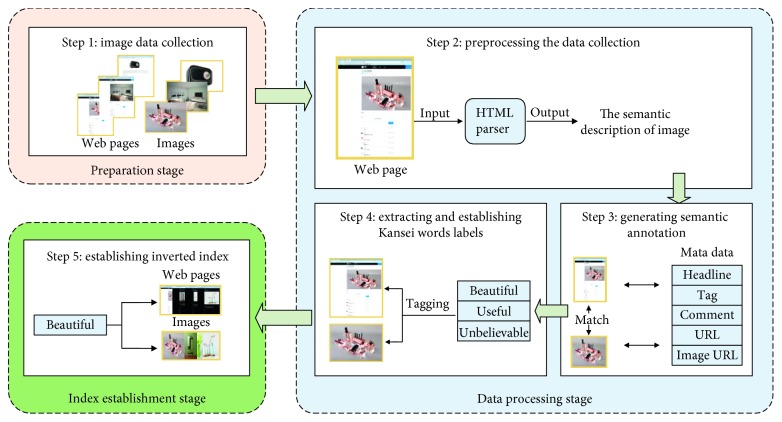
A framework of semantic-based Kansei indexing of inspirational images method.

The method consists of five consecutive steps, as shown in Figure 7, the five steps can be categorized into three stages with respective functionalities. Each of these steps is described below.

#### 4.3.1. Preparation Stage

**Step 1 .**

**Figure 8:**
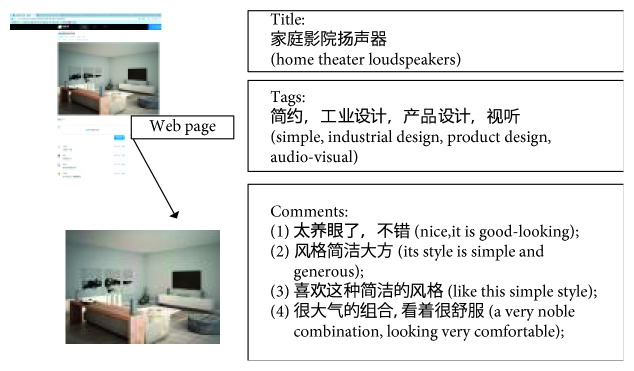
The Kansei evaluation of the description of an image in Pushthink.

Image data collection. Many websites for designs such as Pushthink and CNDESIGN provide a number of images that can inspire designers, including design case images and design material images. Additionally, Kansei evaluations of the semantic description part of these images are performed, such as titles, keywords, image introductions, and comments. These Kansei evaluations are primarily composed of Kansei words (Figure 8). The web crawler is used to crawl the HTML pages of the images and those of these target sites and create a collection of image files and HTML pages.

#### 4.3.2. Data Processing Stage

**Step 2 .**

Preprocessing the data collection. The semantic description of the crawled images in web pages should be further converted into structured data. However, HTML pages contain extensive irrelevant data. For example, in the web page shown in Figure 8, the useful data we need are the images, the URLs of these images, the URL of the HTML, and the Kansei semantic descriptions of the images, specifically in Figure 8, the Kansei semantic description of these images exists in the title, tags, and comments of the HTML page.

As we can know, HTML is semistructured data, and the page structure of the same website is usually fixed. Therefore, after determining the useful data we need, we analyse the structure of the current website and a tool named HtmlParser is used to parse the crawled HTML pages and extract the images, the URLs of HTML pages, URLs of images, and the contents (title, tags, comments, and so on) that contain Kansei semantic description of the images.

**Step 3 .**

Generating semantic annotations for crawled images and web pages. In semantic annotation, one of the important tasks is associating crawled images and web pages with the corresponding semantic description. To do this, we used the following method:

- Firstly, in the process of image data collection, we use the URL in the HTML page of the image as the local storage path to save the crawled image. For example, if the URL of one image in HTML page is “https://assets.pushthink.com/uploads/photo/image/581574/f24724a84f3030e789a979510d0c0793.jpg,” then the storage path of the image on the local disk is “∼/assets.pushthink.com/uploads/photo/image/581574/f24724a84f3030e789a979510d0c0793.jpg”; in this way, each crawled picture is kept in a separate folder.
- Secondly, since the storage path of the image is the same as its URL, the local images are accessed according to their URL parsed from the HTML in Step 2. In order to save the captured image uniformly, a space is allocated on the local disk as the image database, and the accessed images are copied into the image database, and at the same time, MD5 encryption is used to rename every replica image to generate new and unique image URLs for these images copied into the image database.
- Finally, we create a datasheet in MySQL and write the new image URL, HTML URL, and the corresponding image title, keywords, introduction, and comments into the datasheet using JDBC to form a structured storage. In this way, we established the mapping relationships from images to Kansei description.

**Step 4 .**

Extracting and establishing Kansei words labels. Segmentation technology is used to process the Kansei description from Step 3. Using the word list established in KanseiNet to filter the non-Kansei words in the result of the segmentation, the tagging of Kansei words is completed for the crawled images and the source pages of the images.

#### 4.3.3. Index Establishment Stage

**Step 5 .**

Establishing inverted index of the crawled images and the HTML pages of the images. The mapping score indicates the effectiveness of the image mapped to the Kansei word computed. The indexer creates a weighted semantic indexing. Scores are computed using an adaptation of improved TF-IDF algorithm:

(6) $$\begin{array}{c} {\text{Score}}_{\text{tf}}\left({\text{kw}}_{i},i_{n}\right)=\frac{\text{tf}\left({\text{kw}}_{i},i_{n}\right)}{N_{i}}, \end{array}$$

where Score_tf_(kw_*i*_, *i*_*n*_) indicates the score of image in for Kansei word, kw_*i*_, tf(kw_*i*_, *d*_in_)is the word frequency of Kansei word kwi in Kansei word labels of Image *i*_*n*_, and *N*_*i*_ is the number of Kansei word labels of Image *i*_*n*_.

**Figure 9:**
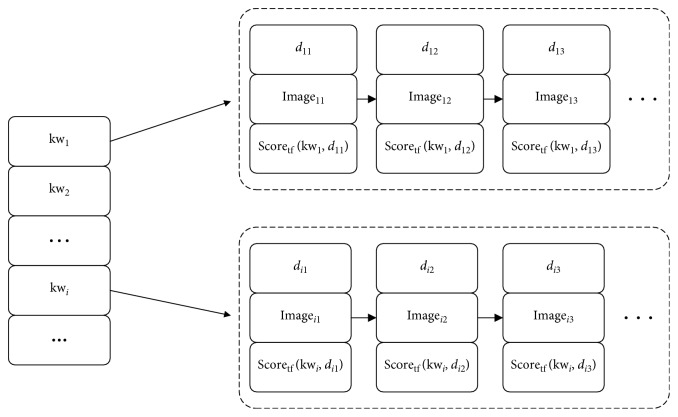
The structure of the established index.

Furthermore, an inverted index of Kansei evaluation for the crawled images and the HTML pages of the images is created. This index is composed of Kansei words, relevant images, and HTML pages. The structure of established index is presented in Figure 9.

## 5. Implementation and Experiment

### 5.1. Prototype System Establishment

Based on the method of retrieval of semantic-based inspirational sources, the Inspirational sources Retrieval System for Emotional Design (SIRSED) is developed.

For establishing the Kansei lexical ontology KanseiNet, the proposed KanseiNet construction method can be used for Chinese and English Kansei words. However, considering the huge difference between Chinese and English and since Chinese Kansei words exhibit greater semantic richness compared to the English Kansei words, the KanseiNet for Chinese is first constructed in this research.

Further, use MyEclipse 2014 as development software; Java as programming language; and JSP, JavaBean, Tomcat, Servlet, Lucene5.0.0, and MySQL5.7.17 as support technologies to achieve the construction of SIRSED. The Browser/Server model is adopted, enabling web users to directly use SIRSED via the browser.

**Figure 10:**
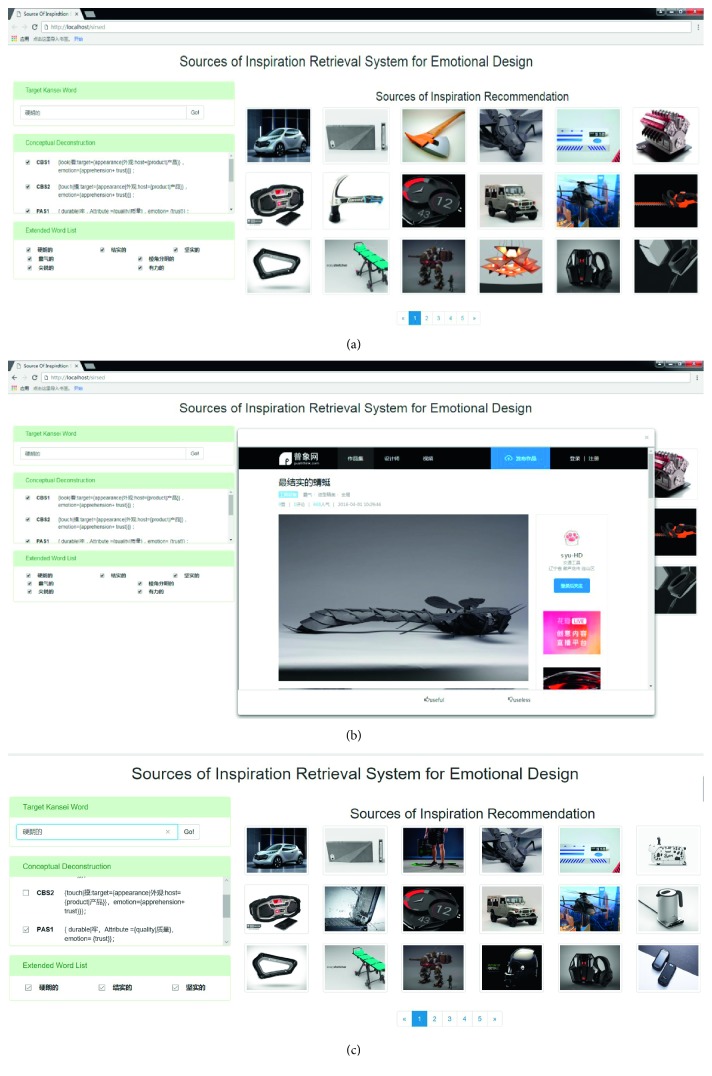
The retrieval interface of SIRSED.

**Figure 11:**
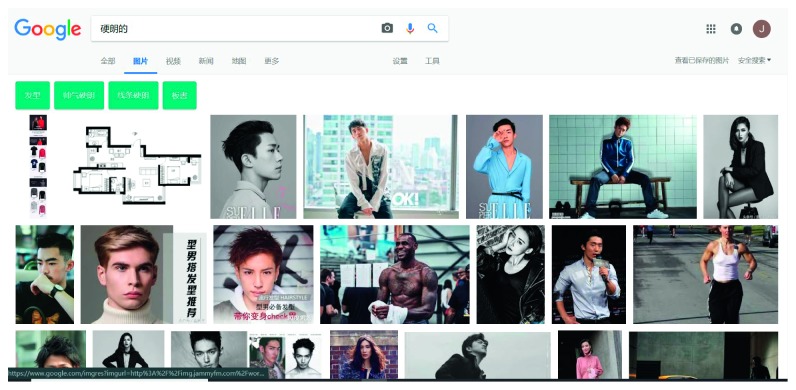
The retrieval results with “硬朗的” in Google Images.

Figures 10 and 11 present the recommended results retrieved with the Chinese Kansei word “硬朗的” in SIRSED and Google Images, respectively, and the English meaning of the Chinese Kansei word “硬朗的” should be “hard” or “tough.” As presented in Figure 10, users click the image in the source page of the image for details.

As shown in Figure 10, an important module in SIRSED is *conceptual deconstruction*. As we described in Section 4.1.2, we determine the similar relationships between the Kansei senses. However, because the designer's actual retrieval needs and different Kansei senses of a Kansei word express different concepts of the Kansei word, in some context, some Kansei senses associated to a specific Kansei word may be more relevant than others. For example, as shown in Figure 10, “硬朗的” has three Kansei senses CBS_1_, CBS_2_, and PAS_1_, designers may prefer to associate with Kansei words which have semantic relationships with Kansei sense PAS_1_ in KanseiNet. To solve this problem, we have adapted a feedback-based approach in SIRSED:

- In the *conceptual deconstruction* module, the designer selects the Kansei senses that he prefers to associate. For example, in Figure 10, the designer only selects the Kansei sense PAS_1_.
- Only the Kansei senses selected by the designer are activated. Since the user only selects the Kansei sense PAS_1_, in the spreading activation process of the Kansei word “硬朗的,” only PAS_1_ is activated, and CBS_1_ and CBS_2_ are not activated.
- Getting an expanded list of words based on the activated Kansei senses. Since only PAS_1_ has been activated, a different expansion result and search result are obtained in Figure 10.

### 5.2. Experiment and Analysis

In order to evaluate whether SIRSED aids designers to search inspirational sources effectively and bridge the semantic gap in emotional design, a verification experiment was carried out.

#### 5.2.1. Experiment

Designers always search for inspirational images with image search engines or in designer-oriented websites. In this experiment, three different systems were used to compare search results, including Google Images search engine, designer-oriented website Pushthink (http://www.pushthink.com), and SIRSED. A total of eight designers participated in this experiment, and ten Chinese Kansei words were taken as query examples, including 硬朗的(hard), 高科技的(high-tech), 创意的(creative), 高贵的(noble), 清爽的(salubrious), 柔软的(soft), 紧凑的(compact), 古典的(classic), 圆润的(mellow), and 温馨的(cosy). Inspirational images could be selected from among the above search results by designers.

#### 5.2.2. Evaluation Metrics

In this study, in order to evaluate the retrieval performance of inspirational sources for emotional design, the recall ratio SI_recall_, precision ratio SI_precision_, and *F*_1_, which is calculated using SI_recall_ and SI_precision_, were introduced in this experiment. The following formulas are used:

(7) $$\begin{array}{c} {\text{SI}}_{\text{recall}}=\frac{N_{\text{rr}}}{N_{\text{rs}}}, \end{array}$$

(8) $$\begin{array}{c} {\text{SI}}_{\text{precision}}=\frac{N_{\text{rr}}}{N_{\text{ss}}}, \end{array}$$

(9) $$\begin{array}{c} F_{1}=\frac{2{\text{SI}}_{\text{recall}}\cdot {\text{SI}}_{\text{precision}}}{{\text{SI}}_{\text{recall}}+{\text{SI}}_{\text{precision}}}, \end{array}$$

where *N*_rr_ is the number of inspirational images retrieved for a Kansei word query, and *N*_rs_ is the total number of inspirational images online for a Kansei word query. However, the total number of inspirational images for a Kansei word query cannot be obtained precisely online. Therefore, the total number of inspirational images for a certain Kansei word query is obtained from the total results using the three above retrieval systems minus the repeat results, after which the recall ratio is calculated using Formula (7). This recall ratio has comparative significance. *N*_ss_ is the total number of images for a Kansei word query. In this experiment, only the first 120 results were used to calculate the precision ratio and the recall ratio. When the number of the results was smaller than 120, the actual results could be used for the calculation.

#### 5.2.3. Results

The results of recall ratio SI_recall_ and precision ratio SI_precision_ from three retrieval systems for ten given Chinese Kansei words are presented in Table 1.

**Figure 12:**
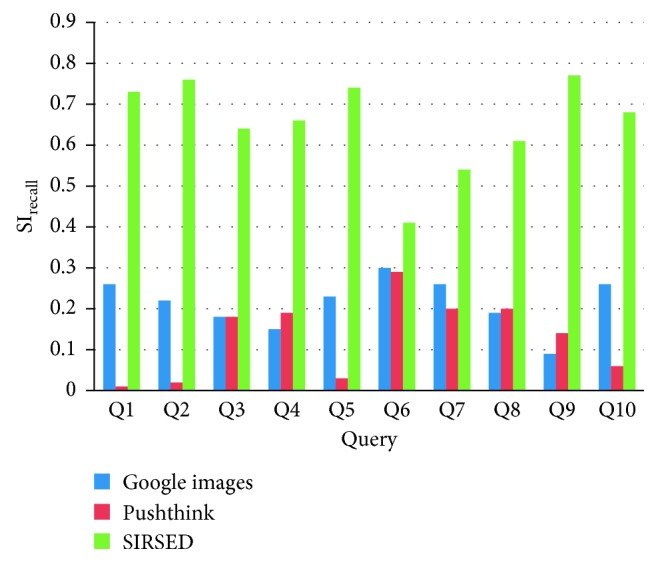
SI_recall_ comparison.

As Figure 12 indicates, the recall ratio SI_recall_ from SIRSED is better than that from the Google Images and Pushthink. The mean recall ratio from SIRSED is 214% higher than that from Google Images and 408% higher than that from Pushthink. One of the reasons for a higher recall ratio of SIRSED is the semantic expansion of Kansei words to aid designer association, and the other is the analysis of the context of images to extract abstract emotional ideas. Therefore, we can infer that compared with existing image retrieval systems, SIRSED can offer more inspirational sources for the same retrieval.

**Figure 13:**
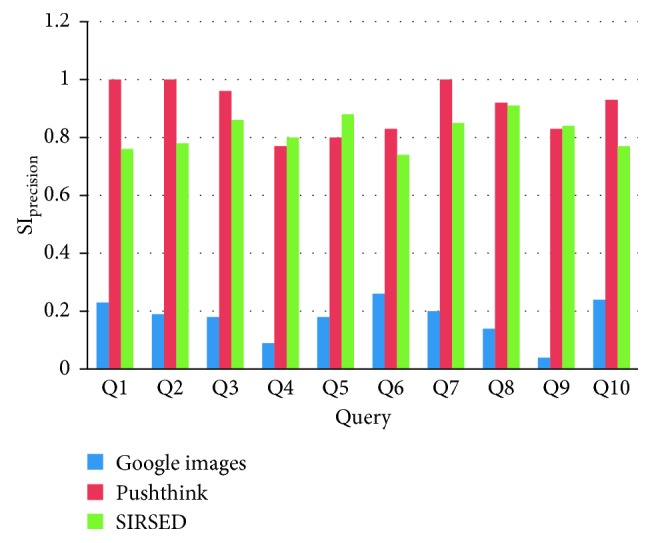
SI_precision_@120 comparison.

As indicated in Figure 13, the high precision ratio SI_precision_@120 is presented in the SIRSED system for ten Kansei words. The retrieval results for four words (noble, salubrious, classic, and mellow) have similar results for SIRSED and Pushthink. The mean precision ratio of SIRSED is 356% higher than that of Google Images and 8% lower than that of Pushthink. Compared with Google Images, the correctness of information sources is focused upon, and the index data are from designer-oriented websites in SIRSED. Therefore, SIRSED is essentially a vertical retrieval of designer-oriented websites based on Kansei semantics. Simultaneously, the index data in Google Images is obtained from different types of websites, and several unnecessary and repeated images are obtained on retrieval. Compared with Pushthink, in SIRSED, the semantic expansions improve the recall ratio of inspirational sources. However, some distant Kansei words could result in irrelevant retrieval results followed by low recall ratio.

**Figure 14:**
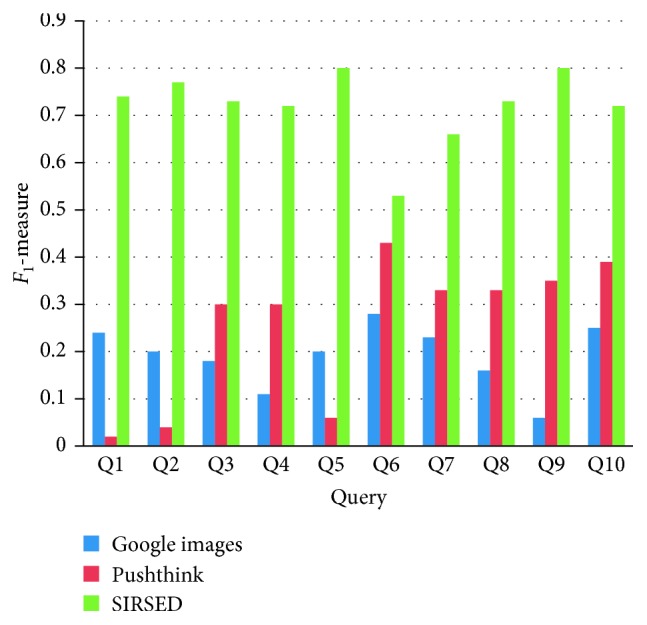
*F*_1_-measure comparison.

The results of *F*_1_ value calculated using SI_recall_ and SI_precision_ are indicated in Figure 14. For ten Kansei words, *F*_1_ from SIRSED is higher than that from Google Images and Pushthink, and indicates that SIRSED performs better.

Overall, the results have revealed that SIRSED is more effective in searching inspirational sources for emotional design and that it is more suitable for supporting designers to obtain inspirational sources in the conceptual design stage of emotional design.

## 6. Conclusion

The study aims to provide abundant inspirational sources during the process of gathering information and idea generation, saving time on gathering information and supporting designers' divergent thinking in emotional design. The highlights are indicated below:

1. The importance of inspirational sources in conceptual design is discussed, and the retrieval requirements of information in emotional design are analyzed and a new method of semantic-based inspirational sources retrieval is proposed.
2. Through conceptual deconstruction of Kansei words and the calculation of the similarity of senses, a Chinese Kansei word lexical ontology KanseiNet is constructed, the SA mechanism based on KanseiNet is proposed, and Chinese Kansei words are semantically extended.
3. The image data of design material are collected and the inverted index of Kansei evaluation for design images is finally established.
4. Based on the retrieval method of semantic-based inspirational sources, SIRSED is developed, and in the experiment comparing with Google Images search and designer-oriented website Pushthink, the SIRSED prototype system is proved to have better pushing performance for emotional design.

The method proposed in this paper provides a new idea for emotional designers to obtain inspirational sources. However, the method has scope for improvement. For example, the weight parameters in the similarity calculation refer to the existing empirical formulas in HowNet. The question arises of how inspirational sources can be provided different stimulation distances through similarity calculation for designers and how it can aid them to generate new ideas with these sources. These above issues should be further investigated in future studies.

## Figures and Tables

**Table 1:** Evaluation results: comparison of Google Images, Pushthink, and SIRSED.

Query	Google Images		Pushthink		SIRSED	
	SI_recall_	SI_precision_	SI_recall_	SI_precision_	SI_recall_	SI_precision_
硬朗的	0.26	0.23	0.01	**1**	**0.73**	0.76
高科技的	0.22	0.19	0.02	**1**	**0.76**	0.78
创意的	0.18	0.18	0.18	**0.96**	**0.64**	0.86
高贵的	0.15	0.09	0.19	0.77	**0.66**	**0.80**
清爽的	0.23	0.18	0.03	0.80	**0.74**	**0.88**
柔软的	0.30	0.26	0.29	**0.83**	**0.41**	0.74
紧凑的	0.26	0.20	0.20	**1**	**0.54**	0.85
古典的	0.19	0.14	0.20	**0.92**	**0.61**	0.91
圆润的	0.09	0.04	0.14	0.83	**0.77**	**0.84**
温馨的	0.26	0.24	0.06	**0.93**	**0.68**	0.77
Mean	0.21	0.18	0.13	**0.90**	**0.66**	0.82

## Data Availability

The experiment data used to support the findings of this study are included within the article.
